# Artificial image objects for classification of breast cancer biomarkers with transcriptome sequencing data and convolutional neural network algorithms

**DOI:** 10.1186/s13058-021-01474-z

**Published:** 2021-10-10

**Authors:** Xiangning Chen, Daniel G. Chen, Zhongming Zhao, Justin M. Balko, Jingchun Chen

**Affiliations:** 1 410 AI, LLC, Germantown, MD 20876 USA; 2 Center for Precision Health, School of Biomedical Informatics, The University of Texas Health Science Center at Houston, Houston, TX 77030 USA; 3 Department of Psychiatry and Behavioral Sciences, McGovern Medical School, The University of Texas, Houston, TX 77030 USA; 4 Department of Medicine, Vanderbilt-Ingram Cancer Center, Vanderbilt University Medical Center, Nashville, TN USA; 5 Breast Cancer Research Program, Vanderbilt-Ingram Cancer Center, Vanderbilt University Medical Center, Nashville, TN USA; 6 Departments of Pathology, Microbiology, and Immunology, Vanderbilt University Medical Center, Vanderbilt-Ingram Cancer Center, Nashville, TN USA; 7 Nevada Institute of Personalized Medicine, University of Nevada Las Vegas, Las Vegas, NV 89154 USA

**Keywords:** RNA sequencing, Breast cancer biomarker classification, Artificial image object, Artificial intelligence, Machine learning algorithm, Convolutional neural network, Image classification

## Abstract

**Background:**

Transcriptome sequencing has been broadly available in clinical studies. However, it remains a challenge to utilize these data effectively for clinical applications due to the high dimension of the data and the highly correlated expression between individual genes.

**Methods:**

We proposed a method to transform RNA sequencing data into artificial image objects (AIOs) and applied convolutional neural network (CNN) algorithms to classify these AIOs. With the AIO technique, we considered each gene as a pixel in an image and its expression level as pixel intensity. Using the GSE96058 (*n* = 2976), GSE81538 (*n* = 405), and GSE163882 (*n* = 222) datasets, we created AIOs for the subjects and designed CNN models to classify biomarker Ki67 and Nottingham histologic grade (NHG).

**Results:**

With fivefold cross-validation, we accomplished a classification accuracy and AUC of 0.821 ± 0.023 and 0.891 ± 0.021 for Ki67 status. For NHG, the weighted average of categorical accuracy was 0.820 ± 0.012, and the weighted average of AUC was 0.931 ± 0.006. With GSE96058 as training data and GSE81538 as testing data, the accuracy and AUC for Ki67 were 0.826 ± 0.037 and 0.883 ± 0.016, and that for NHG were 0.764 ± 0.052 and 0.882 ± 0.012, respectively. These results were 10% better than the results reported in the original studies. For Ki67, the calls generated from our models had a better power for prediction of survival as compared to the calls from trained pathologists in survival analyses.

**Conclusions:**

We demonstrated that RNA sequencing data could be transformed into AIOs and be used to classify Ki67 status and NHG with CNN algorithms. The AIO method could handle high-dimensional data with highly correlated variables, and there was no need for variable selection. With the AIO technique, a data-driven, consistent, and automation-ready model could be developed to classify biomarkers with RNA sequencing data and provide more efficient care for cancer patients.

**Supplementary Information:**

The online version contains supplementary material available at 10.1186/s13058-021-01474-z.

## Background

Breast cancer is a complex disease; early detection and evaluation of the tumor are critical for prognosis and long-term survival. Once a tumor is detected, histopathologic analyses with estrogen receptor, progesterone receptor, human epidermal growth factor receptor2, and evaluation of Nottingham histologic grade (NHG) will be performed. More recently, assessment of the proliferation antigen Ki67 is increasingly recommended [1, 2]. These biomarkers provide valuable prognostic information for survival and treatment outcomes [3, 4]. Therefore, they are used to guide therapeutic strategy selection. However, current approaches to evaluate these biomarkers, i.e., immunohistochemistry stains, require careful assessments by trained pathologists, and disagreements between the pathologists are often observed, especially for NHG and Ki67. Other technical factors, such as sample fixation, antibody batches, and scoring methods, also contribute to the inconsistent results. To obtain a consistent assessment, more robust methods that are amendable to automation are highly desirable.

In recent years, technologies for transcriptome sequencing have become stable and matured, and their applications in clinics are steadily increasing. Some researchers use RNA sequencing to discover new biomarkers; others use it to evaluate existing biomarkers. Although the results vary, the assessment of many markers is comparable to that of histopathologic evaluation. As more and more RNA sequencing data are accumulated, data-driven and machine learning (ML)-based approaches have been explored to discover and classify biomarkers [5–7]. Most of these methods use a variety of strategies to select RNA variants (genes and transcripts) and build classification models. One of the successful examples is the establishment of PAM50 [8], where a collection of expressed genes is used to classify breast cancer into four different subtypes. One key issue in these analyses is the selection of genes and transcripts. This is because many genes are transcribed coordinately; the high correlation between these genes and transcripts, i.e., multicollinearity, makes the selection necessary. Another issue with biomarker discovery and modeling is that most researchers focus on the identification of one, or a limited number, of markers that can be used to predict the outcome measures. This is partially due to the fact that traditional modeling approaches cannot handle a very large number of variants, especially in the case where the number of variants/factors is much larger than the number of observations/sample sizes.

The arise of modern computation power and ML algorithms provides an opportunity to address these issues. Convolutional neural network (CNN) is such an algorithm that has been used very successfully in computer vision and image classification [9, 10]. Recently, CNN algorithms have been applied to classify medical images with exciting results [11–13]. More recently, there are several reports that apply CNN algorithms to the analyses of genomics data [14–16]. We have developed a technology that first transforms tabulated data into artificial image objects (AIOs) and then applies ML algorithms such as CNN to classify these AIOs [17]. In this study, we apply the AIO technique to classify breast cancer biomarkers, with a focus on Ki67 and NHG that disagreements between pathologists are frequently  observed. We hope to demonstrate that a data-driven and ML-based approach could produce consistent assignments for Ki67 and NHG. This report summarizes the results from the study.

## Methods

### RNA sequencing data

We obtained three RNA sequencing datasets from the NCBI GEO database (https://www.ncbi.nlm.nih.gov/geo/), GSE81538 (*n* = 405), GSE96058 (*n* = 2976) [18] and GSE163882 (*n* = 222). The GEO datasets provided pathological assessments for the samples in the datasets and RNA sequencing procedures, which were described previously by the original authors [19]. GSE81538 and GSE96058 were produced by a Swedish team using two sequencing platforms, Hiseq2000 and NextSeq500. GSE163882 was produced by a different team using the NextSeq500 platform. For these datasets, the expression data were measured by Fragments Per Kilobase Million (FPKM). Table 1 summarizes the information of these datasets. The inclusion of GSE163882 was to evaluate the extent to which data produced from a different team could impact on the model performance. After downloading the sequencing data from the GEO Database, a logarithm (log2) transformation was performed for all transcripts; then, the expression levels were rescaled to a range between 0 and 255 for the transcripts. Official gene symbols were extracted from the 3 datasets, and the genes shared among the 3 datasets were used. This procedure generated a list of 16,889 genes. Since most non-coding RNAs did not have official gene symbols, the selected genes were mostly protein coding genes. From this list, we used the first 16,384 genes (genes were sorted by chromosome number and transcription starting position) to create a squared AIO for each of the patients. The 16,384 genes could be configured as a 128 × 128 pixel grayscale AIO or a 64 × 64 × 4 pixel colored AIO.

**Table 1:** Descriptive summary of the datasets used in this study

Dataset	Ki67			NHG				Survival day	Survival event	Sequencing platform
	Ki67−	Ki67+	Missing	Grade I	Grade II	Grade III	Missing			
GSE96058	568	795	1613	449	1394	1074	59	2976	2976	Hiseq2000/NextSeq500
GSE81538	231	174	0	48	167	190	0	0	0	Hiseq2000
GSE163882	0	0	0	17	74	131	0	0	0	NextSeq500

### Clinical data

In this paper, we used the clinical information for Ki67 and NHG to create outcome measures or labels for our model training and prediction. For the Ki67 label, we used the pathologists’ consensus percentage of tumor cells with Ki67 staining to create a binary label. Patients with 20% or less cells stained with Ki67 antibody were assigned as Ki67^−^ or 0; patients with more than 20% of cells stained with Ki67 antibody were assigned as Ki67^+^ or 1. Table 1 summarizes the number of subjects for each category for the GSE81538 and GSE96058 datasets. GSE163882 did not have information on Ki67. NHG had 3 grades, Grades I, II, and III, and they were labeled as 0, 1, and 2 in our model training. For the GSE96058 dataset, there were chemotherapy, endocrine therapy, and survival data that could be used to evaluate the performance of biomarkers. For the GSE163882 dataset, there were only NHG data that could be used to evaluate our model performance.

### Transformation of RNA sequencing data into artificial image objects (AIOs)

**Fig. 1:**
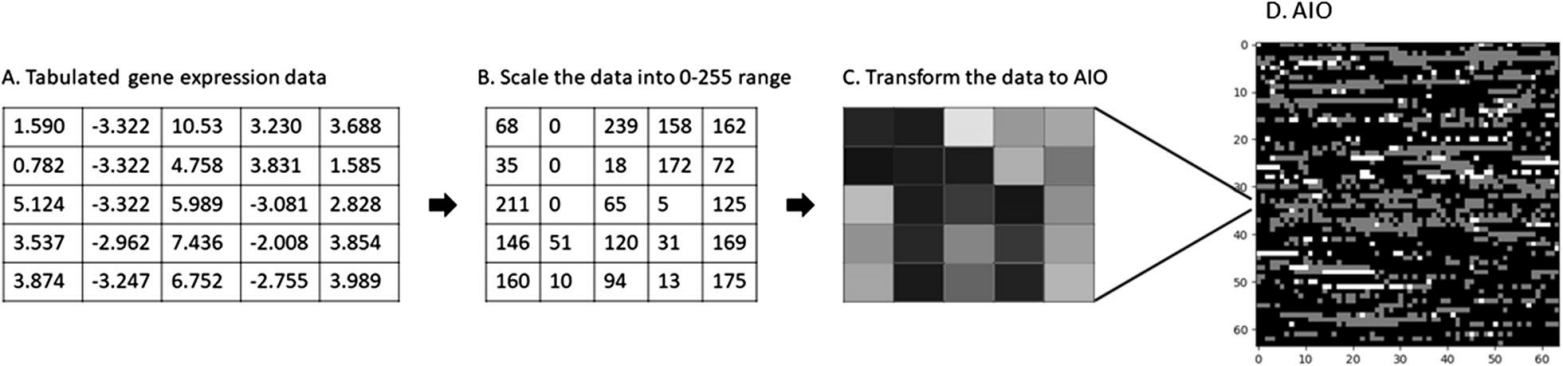
A schematic drawing illustrating the process to transform tabulated gene expression data into AIOs. **a** Tabulated expression data in normalized format. **b** Rescaling the expression data into the range of digital image (1 byte, 0–255). **c** Arranging the expression data from an individual into an artificial image object (AIO). An AIO could be a grayscale image as shown here (**d**) or a colored image in which multiple layers of data could be integrated into an AIO

The AIO technique was based on the concept that considered each element in a dataset, such as a single nucleotide variation in genome wide association study, a gene/transcript in RNA sequencing data, or a CpG locus in methylation study, as a pixel in a digital image so that we could use a collection of elements to create an AIO. With these AIOs, we could apply advanced AI and ML algorithms to analyze and classify them. In this study, we applied this technique to transcriptome sequencing data. When the genes/transcripts were selected, we rescaled the expression levels to a range between 0 and 255 for each gene/transcript. For a given patient, the rescaled expression level would be the pixel intensity of the AIO. From the shared genes among the 3 datasets used in this study, we used the first 16,384 genes (sorted by chromosome number and gene transcription start position) to create an AIO for each of the patients in the datasets. The 16,384 genes could be configured into two different image objects. One was a 128 × 128 (high × wide) pixel grayscale AIO; the other was a 64 × 64 × 4 pixel (high × wide × channel) pseudo-color AIO. The processes to transform gene expression data into grayscale AIOs are shown in Fig. 1. More specifically, for the 128 × 128 configuration, the first 128 genes from the sorted 16,384 list formed the first row of the AIO, and the next 128 genes formed the second row, and so on forward until the last 128 genes formed the last row of the 128 × 128 AIO. For the 64 × 64 × 4 configuration, the first 4096 genes formed the first channel (layer) of the AIO, and the second 4096 genes formed the second channel, and so forth. In these arrangements, the same gene from different individuals occupied the same coordinates on the AIOs, preserving the correlation among the genes as in the original datasets. Therefore, conclusions derived from the classification of the AIOs would be the same as that from the original expression data.

### AIO classification and prediction with convolutional neural network (CNN) algorithms

In this study, we used the TensorFlow (www.tensorflow.org/) [20, 21], Keras (https://keras.io/api/) and the CNN architecture [22, 23] to classify and predict AIOs generated from selected gene expression data. Once the AIOs were made and labels (the calls from trained pathologists) were assigned to the subjects in the 3 datasets, the Tensorflow and Keras platforms were used to conduct image classification analyses. We conducted two sets of analyses. Set I analyses were designed to evaluate how well the whole transcriptome sequencing data could be used to classify and predict the status of Ki67 and NHG. The focus of these analyses was model performance. For this purpose, we combined the GSE96058 and GSE81538 together and used five-fold cross validation with 80–20 splits to evaluate the performance of the models. These analyses were referred to as cross-validation hereafter. Set II analyses were intended to evaluate the generalizability, i.e., how well a model trained with one dataset performed in an independent dataset. For these analyses, we used those subjects with known Ki67 and NHG status from the GSE96058 as training dataset and the subjects from the GSE81538 and GSE163882 as testing datasets. These analyses were referred to as sample testing hereafter. Once the models were trained, we used the models to predict the status of Ki67 and NHG for those subjects with missing information in the GSE96058 dataset (see Table 1). These subjects were then used for survival analyses to compare the predictive performance between model predicted calls and pathologist’s calls.

For the Ki67 binary phenotype, we reported binary accuracy ([true positive + true negative]/[true positive + false positive + true negative + false negative]), precision (true positive/[true positive + false positive]), recall or sensitivity (true positive/[true positive + false negative]), F1 score ([2 × precision × recall]/[precision + recall]) , and the area under the curve (AUC) of the receiver operating characteristic (ROC) for the training process as defined in the scikit-learn package [24]. For the multi-class NHG classification, we reported categorical accuracy and class-specific AUC, precision, recall, and F1 score. Weighted average accuracy and weighted average of AUC were also reported for NHG, which were the sum of the products of class frequency and class-specific accuracy/AUC for each class. For each model, we performed at least 5 runs with slightly different hyperparameters such as learning rate, epsilon value, kernel regularizer values, and kernel size values and reported the mean and standard deviation (sd) for these runs.

### Survival analyses

Survival analyses were conducted with R packages “survival” (https://github.com/therneau/survival) and “survminer” (https://rpkgs.datanovia.com/survminer/index.html), and the results were plotted with R package “ggplot2” (https://ggplot2.tidyverse.org). We compared the predictive performance of Ki67 and NHG status from the pathologist’s calls with that of the calls predicted from our models. The *p* values reported were not corrected for multiple comparisons.

## Results

### Model performance with five-fold cross validation

**Fig. 2:**
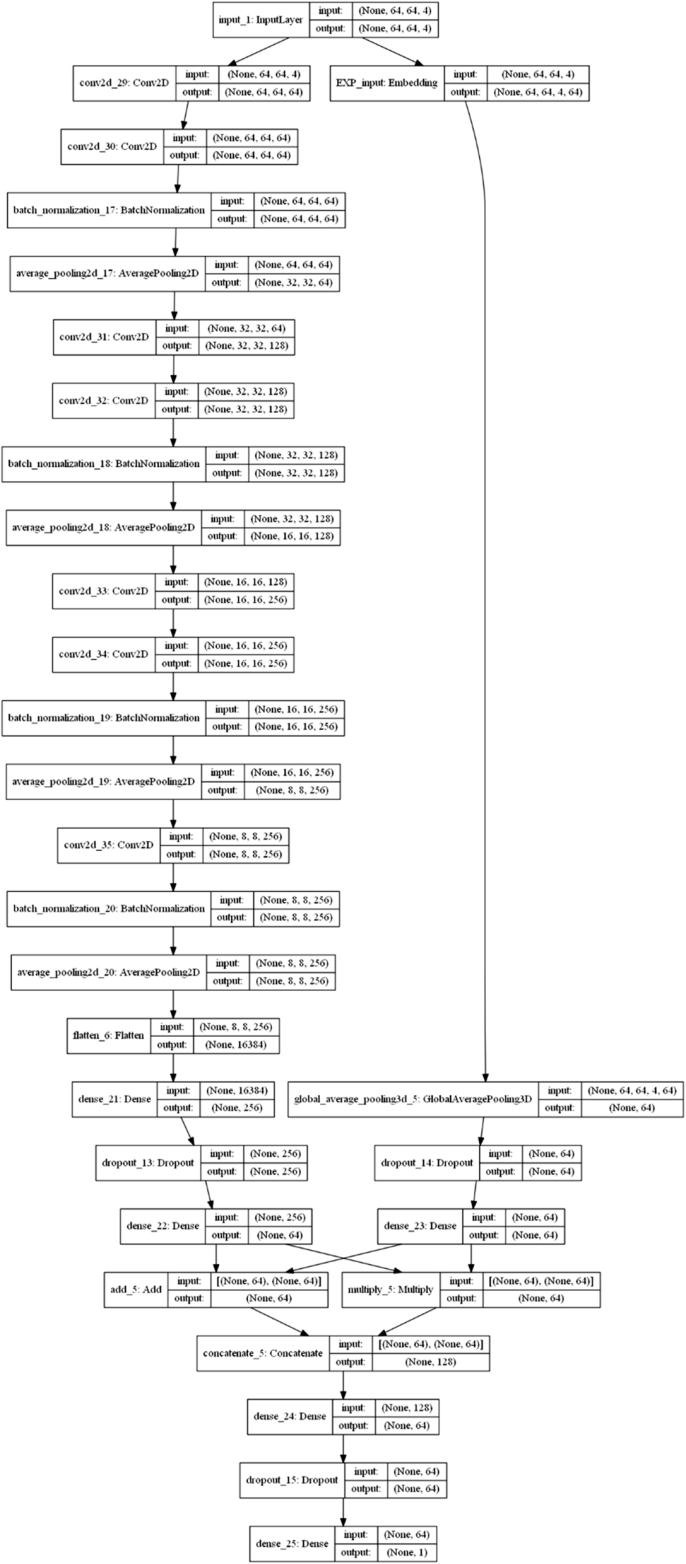
CNN model architecture used for five-fold cross validation. The model had two branches. On the left was a modified VGG structure, and on the right was an embedding layer. The two branches were joined by concatenation before fully connected layers

**Fig. 3:**
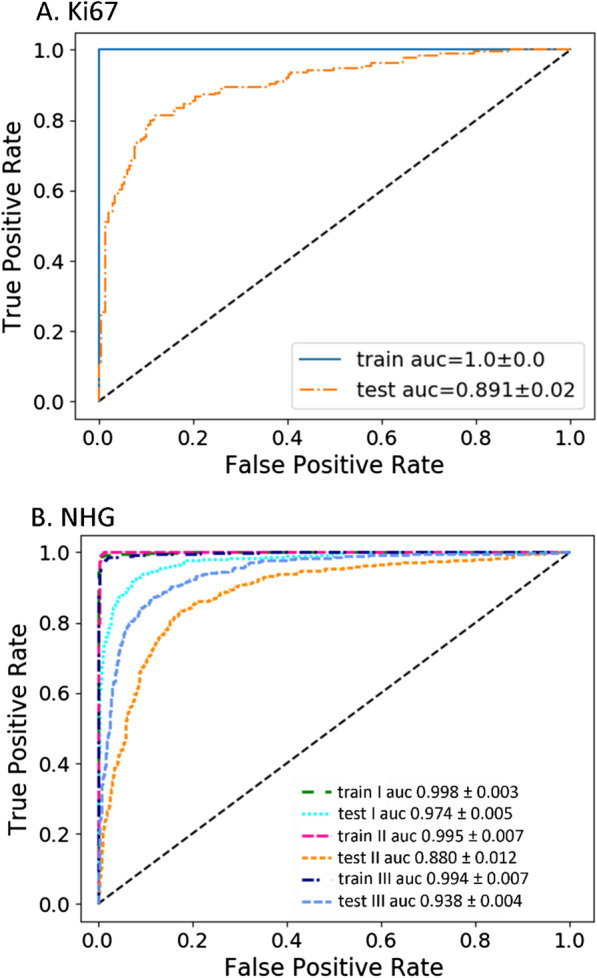
Five-fold cross validation for biomarkers Ki67 and NHG. **a** Ki67, the AUCs of the training and testing samples were shown. **b** NHG, the class-specific AUCs for Grades I, II and III were shown

With the combined GSE96058 and GSE81538 dataset, we tested multiple CNN models to select model hyperparameters, such as the number of convolutional layers, kernel size, regularizer sizes, learning rate, optimizers, and number of fully connected layers. We found that a CNN architecture (Fig. 2) with six 3 × 3 convolutional layers followed with one 1 × 1 convolutional layer and four fully connected layers produced good testing accuracy. The details of the hyperparameters used in the models were included in the Python script posted at our website (https://github.com/mdsamchen/AIO_scripts.git). Figure 3a shows the training and testing AUCs for Ki67, and Table 2 summarizes the detailed results using the 64 × 64 × 4 configuration. For the cross-validation, we obtained a weighted average accuracy of 0.821 ± 0.023 and AUC of 0.891 ± 0.021. The precision, recall and F1 score were 0.822 ± 0.023, 0.822 ± 0.024, and 0.822 ± 0.024, respectively (Table 2).

**Table 2:** Cross-validation and sample testing results for Ki67

	Accuracy	AUC	Precision	Recall	F1-score
*Cross-validation*					
Ki67-			0.815 ± 0.036	0.834 ± 0.022	0.824 ± 0.026
Ki67 +	0.821 ± 0.023	0.891 ± 0.021	0.831 ± 0.016	0.811 ± 0.036	0.820 ± 0.024
Weighted average	0.821 ± 0.023	0.891 ± 0.021	0.822 ± 0.023	0.822 ± 0.024	0.822 ± 0.024
*Sample testing on GSE81538*					
Ki67-			0.886 ± 0.010	0.796 ± 0.048	0.837 ± 0.021
Ki67 +	0.826 ± 0.037	0.883 ± 0.016	0.763 ± 0.036	0.864 ± 0.021	0.809 ± 0.016
Weighted average	0.826 ± 0.037	0.883 ± 0.016	0.833 ± 0.021	0.825 ± 0.036	0.825 ± 0.019

Similar to the Ki67 analyses, we performed five-fold cross validation for NHG as well. Figure 3b shows the class-specific AUCs for the 3 grades, and Table 3 summarizes the results. The performance of NHG was very close to that of Ki67, the weighted average of categorical accuracy was 0.820 ± 0.012, and the weighted average of AUC was 0.931 ± 0.006. The precision, recall, and F1 score were 0.820 ± 0.012, 0.802 ± 0.033, and 0.804 ± 0.030, respectively (Table 3).

**Table 3:** Cross validation and sample testing results for NHG

	Accuracy^a^	AUC^b^	Precision	Recall	F1 Score
*Cross validation*					
Grade I	0.838 ± 0.085	0.974 ± 0.005	0.913 ± 0.028	0.838 ± 0.085	0.871 ± 0.037
Grade II	0.809 ± 0.072	0.880 ± 0.012	0.686 ± 0.07	0.811 ± 0.072	0.738 ± 0.026
Grade III	0.825 ± 0.038	0.938 ± 0.004	0.863 ± 0.031	0.756 ± 0.072	0.803 ± 0.031
Weighted average	0.820 ± 0.012	0.931 ± 0.006	0.820 ± 0.012	0.802 ± 0.033	0.804 ± 0.030
*Sample testing on GSE81538*					
Grade I	0.406 ± 0.081	0.873 ± 0.025	0.608 ± 0.116	0.408 ± 0.082	0.475 ± 0.059
Grade II	0.743 ± 0.069	0.833 ± 0.005	0.710 ± 0.026	0.745 ± 0.070	0.725 ± 0.026
Grade III	0.872 ± 0.029	0.928 ± 0.015	0.848 ± 0.022	0.873 ± 0.030	0.858 ± 0.015
Weighted average	0.764 ± 0.052	0.882 ± 0.012	0.762 ± 0.035	0.765 ± 0.052	0.758 ± 0.025
*Sample testing on GSE163882*					
Grade I	0 ± 0	0.622 ± 0.189	0 ± 0	0 ± 0	0 ± 0
Grade II	0.016 ± 0.006	0.564 ± 0.044	0.268 ± 0.133	0.016 ± 0.006	0.030 ± 0.010
Grade III	0.974 ± 0.012	0.596 ± 0.070	0.589 ± 0.003	0.974 ± 0.012	0.734 ± 0.004
Weighted average	0.580 ± 0.006	0.587 ± 0.038	0.437 ± 0.045	0.330 ± 0.003	0.443 ± 0.004

^a^Categorical accuracy, ^b^class-specific AUC

### Sample testing for the GSE81538 and GSE163882 datasets

In these analyses, we used the portion of the GSE96058 dataset that had Ki67 and NHG status as training samples to classify those patients in the GSE81538 and GSE163882 datasets. The purpose was to evaluate the generalizability of the model using independent training and testing datasets. Based on the results from five-fold cross validation, we made slightly adjustments of the hyperparameters and did 5 or more runs on the GSE81538 and GSE163882 datasets. As shown in Table 2, the performances of sample testing results were similar to that of five-fold cross validation for Ki67 when GSE81538 was used as independent testing dataset. The weighted accuracy for five-fold cross validation was 0.821 ± 0.023; the weighted AUC was 0.891 ± 0.021. In sample testing, the corresponding accuracy and AUC were 0.826 ± 0.037 and 0.883 ± 0.016, respectively (Table 2). For NHG, there were two testing 2 datasets, GSE81538 and GSE163882. For GSE81538, the weighted average of categorical accuracy and class-specific AUC were 0.764 ± 0.052 and 0.882 ± 0.012, respectively (Table 3), slightly worse than that obtained from five-fold cross validation. However, for GSE163882 dataset, which was produced by a different team, the performance was significantly worse (weighted average of categorical accuracy = 0.580 ± 0.006 and weighted average of class-specific AUC = 0.587 ± 0.038). Compared to the multi-gene models reported from the Sweden Cancerome Analysis Network-Breast (SCAN-B) organization[18], the original authors who produced and reported on the GSE81538 and GSE96058 datasets, our AIO approach performed more than 10% better. In their report, the concordance rate or accuracy for Ki67 was 0.663 and that for NHG was 0.667, which were on par with the concordance rates from trained pathologists.

The significantly worse performance of NHG on the GSE163882 dataset surprised us. Therefore, we examined the dataset more carefully. We looked at the correlation of gene expressions, a technical measure of data replicability, among the three datasets used in this study, and we found that the correlation between GSE96058 and GSE81538 was good (Pearson correlation *R* = 0.99), but that between GSE96058 and GSE163882 was moderate (*R* = 0.79), Additional file 1: Fig. S1. This suggested that the poor performance of the GSE163882 on GSE96058 trained models was due to data inconsistency between the training and testing datasets. To further examine whether the structure of the model and the genes used were able to classify NHG, we used the GSE163882 alone and conducted five-fold cross validation with the same model structure. With the same genes and only 222 subjects, we obtained a weighted average accuracy of 0.813 ± 0.026 and weighted average AUC of 0.938 ± 0.007, Additional file 1: Table S1. The results were virtually the same as that of the combined GSE96058 and GSE81538 dataset, suggesting that the structure of the model and the selected genes was capable to classify NHG.

### Comparison between AIO configurations and CNN architectures

The results above for both Ki67 and NHG were obtained with AIOs using the 64 × 64 × 4 configuration. To evaluate whether and to what extent that AIO configuration influenced model performance, we did sample testing with the 128 × 128 configuration for the two markers. The overall performances between the two configurations were similar for both Ki67 and NHG (comparing Table 2 with Additional file 1: Table S2, and Table 3 with Additional file 1: Table S3). For example, the weighted average accuracy for Ki67 was 0.826 ± 0.037 for the 64 × 64 × 4 configuration (Table 2), that for the 128 × 128 configuration was 0.825 ± 0.012 (Additional file 1: Table S2). Similarly, the weighted average of accuracies for NHG for the two configurations were 0.764 ± 0.052 and 0.766 ± 0.009, respectively. These results suggested that the configuration of the AIOs had minimal influence on model performance.

We also compared the impact of CNN architectures on classification performances. All results, up to this point, were obtained with a two-dimension convolution CNN architecture (2D-CNN). Since genes were arranged on chromosomes linearly, we could treat gene expression as an one-dimensional data such that we could use an one-dimension convolution CNN architecture (1D-CNN) to classify the biomarkers. The results were summarized in Additional file 1: Tables S4 and S5. For Ki67, the weighted average accuracy and AUC for 1D-CNN were 0.803 ± 0.054 and 0.851 ± 0.019, which were very close to the results obtained with 2D-CNN (0.826 ± 0.037 and 0.883 ± 0.016). For NHG, the weighted average accuracy and weighted average of AUC were 0.766 ± 0.009 and 0.880 ± 0.011 with 1D-CNN, and 0.764 ± 0.052 and 0.882 ± 0.012 with 2D-CNN, respectively. Again, we found that the differences between 1D-CNN and 2D-CNN were minimal.

### Survival analyses for GSE96058 and GSE81538 subjects

**Fig. 4:**
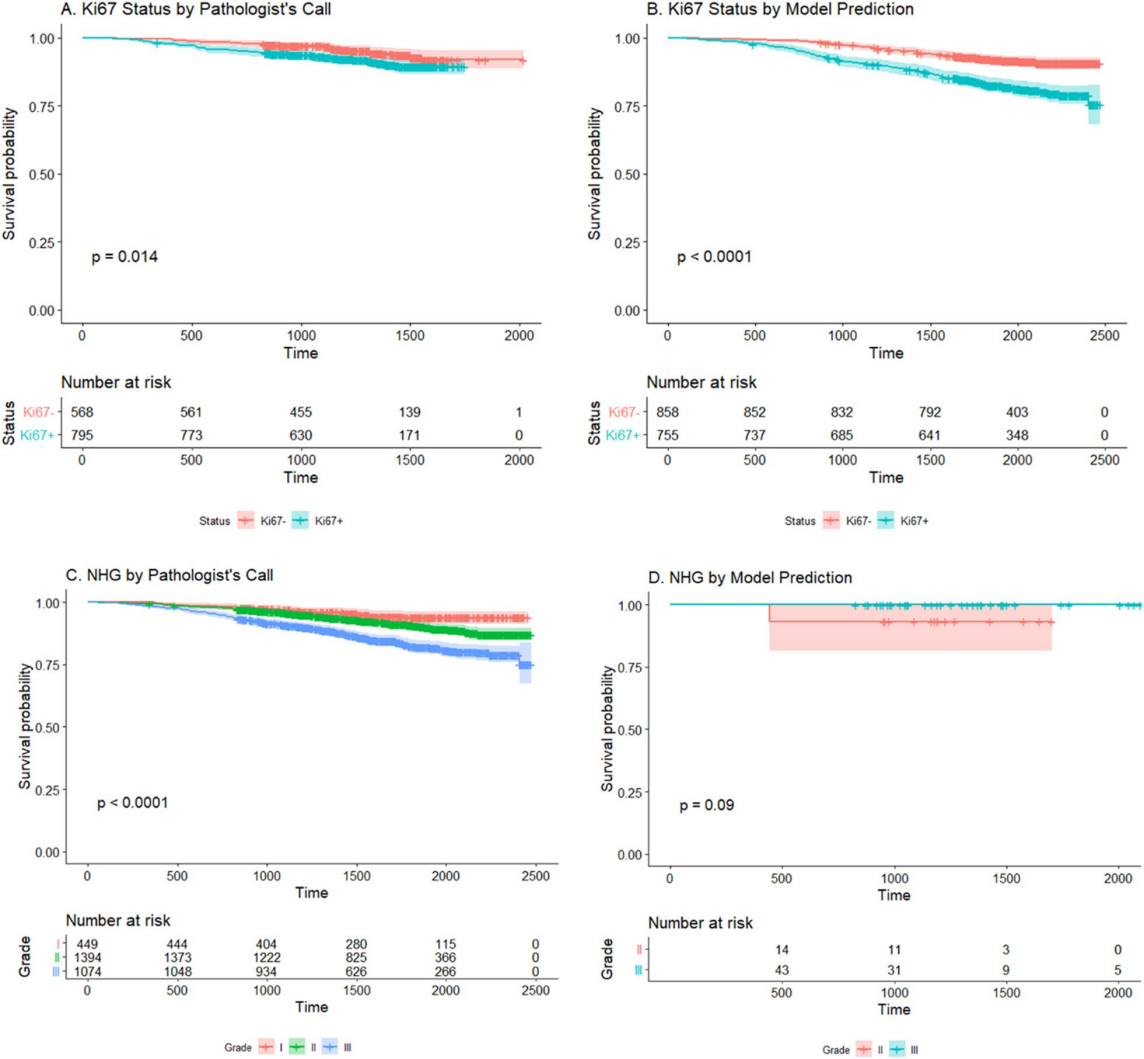
Comparison of performance between pathologists’ consensus calls and model produced calls. **a** Survival analyses for pathologist’s consensus calls of Ki67 status. **b** Survival analyses for model produced calls of Ki67 status. **c** Survival analyses for pathologist’s consensus calls of NHG. **d** Survival analyses for model produced calls of NHG. For Ki67, the calls from the model had better performance in survival analyses than that of pathologist’s consensus calls. For NHG, the performance of model produced calls only showed a trend, this was likely due to the much smaller sample size (*N* = 59 as compared to *N* = 2917 from the pathologist’s calls, see Table 1)

The value of biomarkers in the care of breast cancer patients was their ability to predict treatment outcomes and survival rate. In addition to measure model accuracy and AUC, another approach to evaluate the performance of the models was to test whether the calls produced  from the models had similar predictive power as the calls obtained  from trained pathologists. Based on this rationale, we conducted comparative analyses using the subjects in the GSE96058 dataset. In the GSE96058 dataset, there were 1363 subjects with pathologist assigned status for Ki67, and there were 1613 subjects with missing data for Ki67 status (Table 1). We used our model to predict the Ki67 status for those subjects with missing status and conducted survival analyses for these two groups of subjects (Fig. 4). Comparing Fig. 4a with b, we could see that the performance of model produced  calls was better than that of the calls from trained pathologists, suggesting that with our CNN model, we could classify Ki67 status and achieve a better predictive power in survival analyses than that obtained  by trained pathologists (*p* value < 0.0001 vs *p* value = 0.014). We conducted similar analyses for the NHG. Since the number of subjects with missing NHG status was very small (*n* = 59) compared to the subjects with pathologist assigned NHG status (*n* = 2917), we only saw a trend (*p* value = 0.09) for the model produced calls (Fig. 4d).

## Discussion

With the advancement of high-throughput DNA sequencing technologies, transcriptome sequencing had been used increasingly in clinical studies. The rapid accumulation of large transcriptome data presented a great opportunity to apply ML algorithms to address clinical issues. In this study, we adopted the CNN algorithms to breast cancer RNA sequencing data and developed models to classify two commonly used biomarkers. Our goals were twofold: first, to evaluate the application of the AIO technique to RNA sequencing data, and second, to evaluate the performance of our CNN models with other methods that were currently used for the classification of breast cancer biomarkers. The reason we focused on Ki67 and NHG was that the assessments for these markers by pathologists were not very consistent, improvement in prediction accuracy was of high clinical value.

We designed two sets of experiments to evaluate how the combination of AIO technique and CNN algorithms performed with RNA sequencing data for biomarker classification. In the first experiment, we used cross validation techniques to assess the models built with the AIO technique and CNN algorithms. Here we combined the GSE96058 and GSE81538 datasets and performed five-fold cross validation for both Ki67 and NHG markers. For Ki67, a binary classification, we accomplished an accuracy of 0.821 ± 0.023 and AUC of 0.891 ± 0.021 (Table 2). The precision, recall, and F1 score were 0.822 ± 0.023, 0.822 ± 0.024, and 0.822 ± 0.024, respectively. For NHG, a multi-class classification, the weighted average of categorical accuracy and the weighted average of class-specific AUC were 0.820 ± 0.012 and 0.931 ± 0.006 (Table 3).

In the second experiment, sample testing, we used GSE96058 as training dataset to build the model and tested its performance with independent GSE81538 and GSE163882 datasets. We used GSE96058 as training data because it had much larger sample size (*n* = 2976) compared to that of GSE81538 (*n* = 405). Both GSE96058 and GSE81538 were produced by a Swedish team [18, 19], and GSE163882 was produced by a different team. For Ki67, the Swedish team reported a multi-gene model with an accuracy of 0.663 as compared to the consensus calls from trained pathologists. Our model reported an accuracy of 0.826 (Table 2). For NHG, the Swedish team reported an accuracy of 0.677 and our CNN model reported an accuracy of 0.764 (Table 3). For GSE163882, our model trained with GSE96058 did not perform well, with weighted accuracy of 0.580 ± 0.006 and weighted AUC of 0.587 ± 0.038, respectively. To find the reason why GSE163882 did not perform well, we looked at the correlation of gene expressions between GSE96058 and GSE163882, and we found that the correlation was not very good (Pearson correlation coefficient, *R* = 0.79). As a comparison, the correlation of gene expressions between GSE96058 and GSE81538 was very good (*R* = 0.99) (Additional file 1: Fig. S1). These analyses indicated that the poor performance of GSE163882 was, at least in part, due to the data inconsistency between the GSE96058 and GSE163882 datasets. While both datasets were produced with Illumina NextSeq500 platform, the technical details how the sequencing was conducted could vary significantly. Furthermore, GSE96058 and GSE163882 used a different sample treatment. GSE96058 used freshly frozen tissue samples, and GSE163882 used formalin-fixed paraffin-embedded samples. All these contributed to the inconsistent sequencing data. While the models trained with GSE96058 did not perform well on GSE163882, cross validation using only the GSE163882 data with the same genes and CNN architecture did produce comparable accuracy (0.813 ± 0.026) and AUC (0.938 ± 0.007) as that of the combined GSE96058 and GSE81538 dataset (compare Additional file 1: Table S1 and Table 3). These results indicated that by transforming gene expression data into AIOs, we could apply mature algorithms such as CNN to effectively classify biomarkers and accomplish comparable or better accuracy as compared to other modeling methods. However, the significant performance differences between GSE81538 and GSE163882 datasets stressed the importance of data consistency. For a model to have good generalizability and to be used in clinical applications, it was critically important to establish stable and consistent pipelines for data production. The different performance of NHG between the GSE81538 and GSE163882 datasets made it clear of this principle that was well documented in the literature.

We evaluated how AIO configurations and CNN architecture impacted on Ki67 and NHG classification. The 16,384 genes could be configured into two different image objects. For both grayscale 128 × 128 and pseudo-color 64 × 64 × 4 configurations, our models produced similar classification accuracies (see Tables 2, 3, Additional file 1: Tables S2 and S3). This was consistent with the results obtained from classification of black-white and colored images in the field of computer vision. But for the AIO technique, this was significant because this suggested we could put different types of genomics data into separate layers/channels and incorporated them into an AIO, allowing integrated analyses with multi-omics data. We also built models to classify Ki67 and NHG using one-dimensional convolution, because genes were aligned linearly on chromosomes, and conceptually, they could be considered one-dimensional data suitable for one-dimensional convolution analyses [15]. In our analyses, 1D-CNN and 2D-CNN produced comparable results (compare Tables 2 and 3 with Additional file 1: Tables S4 and S5). Typically, 1D-CNN was used to analyze one-dimensional data, such as time series, audio, text, and electrocardiogram data [25], and 2D-CNN was used for computer vision and image classification. While there were techniques transforming one-dimensional data into images for 2D-CNN analyses [26], we were not aware of extensive analyses to compare the performances between 1D-CNN and 2D-CNN using the same data. With our limited analyses, it would be difficult to conclude whether one approach was better than the other. Researchers should explore both if the data could be analyzed with these algorithms.

We evaluated the predictive power of the calls produced from our CNN models by comparing it to that of the consensus calls from trained pathologists. With similar sample size (see Table 1), the Ki67 calls produced from our models had a better power in survival analyses than the calls from trained pathologists (Fig. 4a, b). The reason for this might be that model produced calls were more consistent than human raters. For NHG, with a very small sample size (*n* = 59), the model produced calls showed a trend. Should we have a larger sample size, the model produced calls would produce significant results. These results demonstrated that once implemented, these models would improve the productivity and consistency in clinical applications.

In the literature, there was a report that proposed a different method, DeepInsght, to transform non-image data into images for CNN classification [16]. Both our AIO technique and the DeepInsight enabled the application of CNN algorithms to non-image data and had the potential to apply to large and multiple datasets. But there was a key difference between our AIO technique and the DeepInsight. For the AIO technique, we considered each feature/variable as a pixel, and directly mapped the features onto the feature map. In contrast, the DeepInsight approach first performed a kernel PCA/tSNE transformation to determine the relationship among the features and then used this information to determine the coordinates of the features on the image. After kernel PCA or tSNS transformation, not only it could cause substantial loss of information, but also created a situation where multiple features mapped to the same coordinates on the transformed image. This made it very difficult to track which features contributed the most to the patterns that the CNN algorithms learned and used to classify the label, a piece of information that could be significant for understanding the underlying biology when genomics data were used. This ability to track the genes in the spatial patterns provided a new approach to discover multi-gene interactions and interaction networks. Given the differences between the two methods, it would be interesting to compare their performances using the same datasets. Overall, both methods would have broad applications in biomedical research.

## Conclusions

In this article, we reported the application of a technique to transform genomic data into AIOs and adopted CNN algorithms for their classification. Applying the technique to Ki67 and NHG, biomarkers that had substantial inconsistent assessments among trained pathologists, we demonstrated that our CNN models achieved classification accuracies better than similar models reported in the literature. Furthermore, with survival analyses, we showed that the calls generated by the models had a better predictive power than the consensus calls made by trained pathologists. These results illustrated the utility of the AIO technique in biomarker classification. With the demonstration of the principle, the AIO technique could have broad applications in clinical and genomics studies, facilitating more effective care of cancer patients.

## Supplementary Information

**Additional file 1**. Supplementary information.

## Data Availability

Not applicable.
