# Supplementary material for: Artificial image objects for classification of breast cancer biomarkers with transcriptome sequencing data and convolutional neural network algorithms
Source: Breast Cancer Res. 2021 Oct 10;23:96. doi: 10.1186/s13058-021-01474-z (PMC8504079; doi:10.1186/s13058-021-01474-z)
Supplement: Supplementary file 1 — Additional file 1. Supplementary information. [file 13058_2021_1474_MOESM1_ESM.docx]

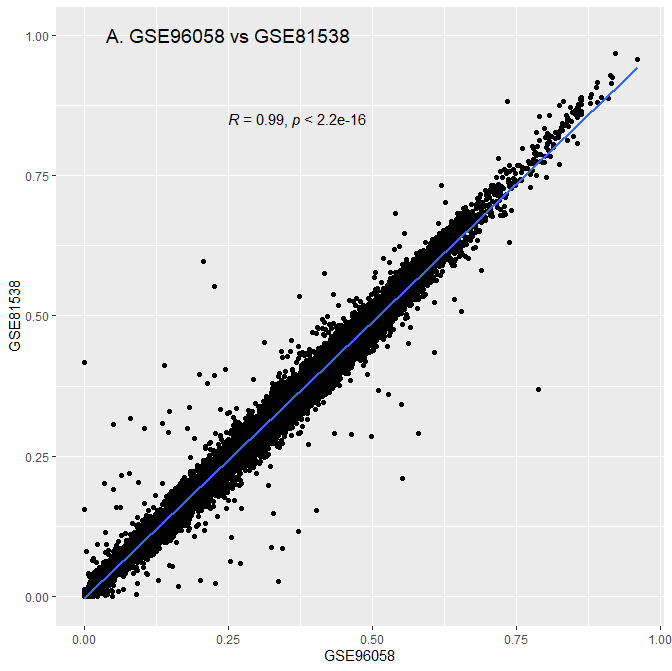


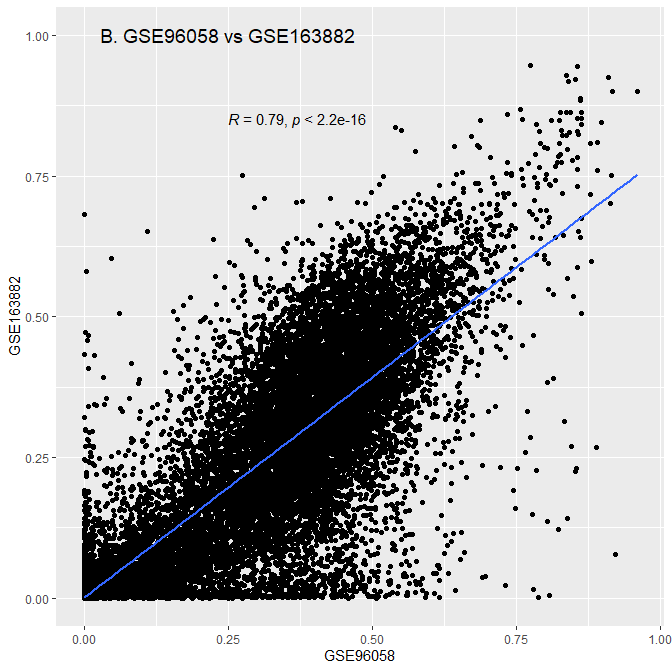


**Figure S1. Gene expression correlation among the GSE96058, GSE81538 and GSE163882 datasets.**

A. Pearson correlation between the GSE96058 and GSE81538 datasets. B. Pearson correlation between the GSE96058 and GSE163882 datasets. For both panels, the means of the gene expression between the two datasets were plotted.

**Table S1. Five-fold cross-validation with GSE163882 dataset**

|  | **Accuracy^1^** | **AUC^2^** | **Precision** | **Recall** | **F1 Score** |
| --- | --- | --- | --- | --- | --- |
| Grade I | 0.963 ± 0.008 | 0.992 ± 0.004 | 0.944 ± 0.021 | 0.963 ± 0.008 | 0.953 ± 0.01 |
| Grade II | 0.719 ± 0.053 | 0.892 ± 0.016 | 0.732 ± 0.07 | 0.719 ± 0.053 | 0.722 ± 0.021 |
| Grade III | 0.742 ± 0.102 | 0.924 ± 0.011 | 0.754 ± 0.021 | 0.742 ± 0.102 | 0.745 ± 0.051 |
| **Weighted average** | **0.813 ± 0.026** | **0.938 ± 0.007** | **0.815 ± 0.023** | **0.808 ± 0.022** | **0.812 ± 0.022** |

1: Categorical accuracy; 2: Class specific AUC.

**Table S2. Sample testing of GSE81538 with the 128 × 128 configuration for Ki67**

|  | **Accuracy** | **AUC** | **Precision** | **Recall** | **F1-score** |
| --- | --- | --- | --- | --- | --- |
| Ki67- | 0.909 ± 0.013 |  | 0.902 ± 0.030 | 0.718 ± 0.035 | 0.798 ± 0.020 |
| Ki67+ | 0.713 ± 0.030 | 0.883 ± 0.036 | 0.706 ± 0.025 | 0.894 ± 0.038 | 0.786 ± 0.013 |
| **Weighted average** | **0.825 ± 0.012** | **0.883 ± 0.036** | **0.818 ± 0.028** | **0.794 ± 0.036** | **0.793 ± 0.017** |

**Table S3. Sample testing of GSE81538 with the 128 × 128 configuration for NHG**

|  | **Accuracy** | **AUC** | **Precision** | **Recall** | **F1 Score** |
| --- | --- | --- | --- | --- | --- |
| Grade I | 0.388 ± 0.046 | 0.810 ± 0.017 | 0.611 ± 0.030 | 0.388 ± 0.046 | 0.472 ± 0.029 |
| Grade II | 0.760 ± 0.046 | 0.768 ± 0.004 | 0.705 ± 0.014 | 0.760 ± 0.046 | 0.731 ± 0.018 |
| Grade III | 0.866 ± 0.026 | 0.876 ± 0.005 | 0.849 ± 0.024 | 0.866 ± 0.026 | 0.857 ± 0.008 |
| **Weighted average** | **0.766 ± 0.009** | **0.815 ± 0.007** | **0.748 ± 0.020** | **0.753 ± 0.038** | **0.746 ± 0.015** |

**Table S4. Sample testing of GSE81538 with 1D CNN architecture for Ki67**

|  | **Accuracy** | **AUC** | **Precision** | **Recall** | **F1-score** |
| --- | --- | --- | --- | --- | --- |
| Ki67- | 0.852 ± 0.056 |  | 0.852 ± 0.055 | 0.780 ± 0.058 | 0.814 ± 0.009 |
| Ki67+ | 0.738 ± 0.030 | 0.851 ± 0.019 | 0.738 ± 0.031 | 0.812 ± 0.093 | 0.770 ± 0.028 |
| **Weighted average** | **0.803 ± 0.054** | **0.851 ± 0.019** | **0.803 ± 0.045** | **0.794 ± 0.073** | **0.795 ± 0.017** |

**Table S5. Sample testing of GSE81538 with 1D CNN architecture for NHG**

|  | **Accuracy** | **AUC** | **Precision** | **Recall** | **F1 Score** |
| --- | --- | --- | --- | --- | --- |
| Grade I | 0.363 ± 0.081 | 0.876 ± 0.021 | 0.635 ± 0.056 | 0.363 ± 0.081 | 0.456 ± 0.071 |
| Grade II | 0.762 ± 0.035 | 0.836 ± 0.009 | 0.708 ± 0.022 | 0.762 ± 0.035 | 0.733 ± 0.009 |
| Grade III | 0.872 ± 0.042 | 0.936 ± 0.011 | 0.839 ± 0.017 | 0.872 ± 0.042 | 0.854 ± 0.016 |
| **Weighted average** | **0.766 ± 0.009** | **0.880 ± 0.011** | **0.749 ± 0.024** | **0.752 ± 0.044** | **0.744 ± 0.020** |
